# ‘Multi-omics’ data integration: applications in probiotics studies

**DOI:** 10.1038/s41538-023-00199-x

**Published:** 2023-06-05

**Authors:** Iliya Dauda Kwoji, Olayinka Ayobami Aiyegoro, Moses Okpeku, Matthew Adekunle Adeleke

**Affiliations:** 1 Discipline of Genetics, School of Life Sciences, College of Agriculture, Engineering and Sciences, University of KwaZulu-Natal, 4090 Durban, South Africa; 2 Unit for Environmental Sciences and Management, North-West University, Potchefstroom, Northwest South Africa

**Keywords:** Bacterial genetics, Genomics

## Abstract

The concept of probiotics is witnessing increasing attention due to its benefits in influencing the host microbiome and the modulation of host immunity through the strengthening of the gut barrier and stimulation of antibodies. These benefits, combined with the need for improved nutraceuticals, have resulted in the extensive characterization of probiotics leading to an outburst of data generated using several ‘omics’ technologies. The recent development in system biology approaches to microbial science is paving the way for integrating data generated from different omics techniques for understanding the flow of molecular information from one ‘omics’ level to the other with clear information on regulatory features and phenotypes. The limitations and tendencies of a ‘single omics’ application to ignore the influence of other molecular processes justify the need for ‘multi-omics’ application in probiotics selections and understanding its action on the host. Different omics techniques, including genomics, transcriptomics, proteomics, metabolomics and lipidomics, used for studying probiotics and their influence on the host and the microbiome are discussed in this review. Furthermore, the rationale for ‘multi-omics’ and multi-omics data integration platforms supporting probiotics and microbiome analyses was also elucidated. This review showed that multi-omics application is useful in selecting probiotics and understanding their functions on the host microbiome. Hence, recommend a multi-omics approach for holistically understanding probiotics and the microbiome.

## Introduction

The gastrointestinal tract (GIT) is an intricate ecosystem harboring the microbiome consisting of fungi, bacteria, viruses and archaebacteria living symbiotically with the host^1^. The microbiome benefits the host through selective carbohydrates and polyphenols fermentation^2^ to produce bioactive metabolites^3^. However, age, diet, antimicrobials, and stress may cause dysbiosis contributing to chronic diseases. Probiotics, the preparations of microorganisms with health benefits when consumed by the host in adequate amounts^4^, have been used to modulate and restore the gut microbiome, stimulate immune response and enhance the host’s resistance to diseases^5,6^. Increasing awareness regarding the microbiome has greatly improved the consideration of microbes beyond disease-causing pathogens to a better understanding of their beneficial effects on human and animal health, thereby expanding the use of probiotics^7,8^. Formerly, the knowledge of probiotics was limited to elementary microbiology and food processes. However, this has changed in the postgenomic period of biomedicine as an important area for developing functional nutraceuticals, gastroenterology, allergology, skin care, cancer therapy, psycho-neuroendocrinology, and veterinary applications^9^. Probiotics have received increased attention in the scientific, healthcare settings, and the larger society^10^. Lactic acid bacteria (LAB), *Bifidobacterium*, *Escherichia coli* Nissle1917, and yeasts (*Saccharomyces cerevisiae* and *S. boulardii*) are among the species of microbes regarded as safe status with the ability to express heterologous genes encoding anti-inflammatory and antimicrobial biomolecules^9^. Other potential bacteria, such as *Akkermansia muciniphila*, with the ability to improve the host metabolic function and immunity during cancer treatment^11^, and *Faecalibacterium prausnitzii* with benefits, including the improvement of liver health by regulating fat build-up^12^ and alleviates atopic dermatitis in experimental animals were identified and termed next-generation probiotics (NGPs)^13^. The growing awareness of the application of beneficial microbes and the increase in data generation due to the characterization of these microbes have necessitated a clearer understanding of probiotics for precision therapy because they are strain^14^, disease^15^, and host specificity in their actions^16^. Therefore, the application of multi-omics will grant a holistic understanding of the mechanism of probiotic action at the system biology level for efficient applications.

The biological system has many regulatory features, including DNA, mRNA, proteins, metabolites, and epigenetic components such as DNA methylation and histone post-translational modifications (PTMs). These features can influence the signaling cascades and phenotypes when affected by physiological or pathological changes. Furthermore, the microbiome can influence the host’s genome, protein expression, and PTMs^17^. Hence, the advent of high-throughput technologies, including whole genome, transcriptome, and reduced representation bisulfite sequencing, and liquid/gas chromatography-mass spectrometry (LC-MS, GC-MS) have greatly enhanced the comprehensive studies of molecular features at different omics levels^18^. A system biology approach permits the integration of large datasets from genomics, epigenomics, transcriptomics, proteomics, metabolomics, and lipidomic (the so-called “multi-omics”) analyses from experimental and theoretical models^19^. Technological advancement, including DNA and RNA sequencing, proteomics, metabolomics, lipidomics, and the initiation of microbiome studies and computational collation of clinical and research data in the past two decades, has increased biological data generation. These developments require advanced analytical tools to derive useful biological information for meaningful inferences^19^. Omics tools are continually becoming powerful methods of obtaining an impartial and integrated view of complex biological processes such as the course of diseases and treatment efficiency^20,21^. Multi-omics data analysis derives valuable information about cellular functions^21^ and grants an understanding of the complex biology^22^ with a clear picture of the endotypes^23^. This review describes the growing aspects of system biology using multi-omics related to probiotic studies while elucidating its effects on the host and the gut microbiome.

## Omics techniques used in studying probiotics and microbiome

### Genomics and metagenomics

The development of ‘omics’ technologies has changed the perspective in research by generating high-throughput genomic data, bridging genome and transcription to the phenome^24^. An organism’s genome assembly is necessary for understanding its biology. The genomics revolution in microbiology has enabled the sequencing of diverse strains of microbial species with clear information on the pangenome for protein-coding sequences^25^. For example, accurate genome sequencing demonstrates several differences between microbial reference sequences of a species^26^ with considerable physiological effects that can influence experimental results with downstream consequences on bioprocess designs^27^. The use of whole-genome sequencing to decipher the activities of *Limosilactobacillus reuteri* PNW1 (formerly; *Lactobacillus reuteri* PNW1) showed several genes in the genome assembly that are important for its action as a probiotic^28^. Alayande et al.^28^ further revealed the presence of several important genes, including those associated with lactic acid production, mucosal adhesion, stress tolerance, and therapeutically useful peptides (Table 1). Data from genomic analyses are also useful in tracing the origin of probiotic bacteria and their relationship with the gut microbiome. Pasolli et al.^29^ applied comparative genomics analyses to show the diversity and relationship of the LAB strains present in food and gut microbiome, with fermented food as the frequent niche of LAB in nature. Another comparative genomics study of the lactobacilli genomes revealed extensive gene loss and acquisitions via horizontal transfer during co-evolution in their habitats^30^.

**Table 1:** ‘Omics’ techniques applied in studying probiotic and microbiome interaction.

Tool	Probiotic bacteria	Application	Findings	References
Whole-genome sequencing	*Limosilactobacillus reuteri* PNW1	Determination of probiotic potential	• Genes encoding D and L-lactate dehydrogenases. • Genes responsible for adhesion to epithelial tissues, including antiadhesion Pls, Sortase A, exopolysaccharide cluster, • Genes encoding bioactive peptides (S-ribosyl homocysteine lyase, Autoinducer-2 production protein *LuxS* • Four coding regions associated with enhancing host metabolism and enzymes such as Poly (glycerol-phosphate) alpha-glucosyltransferase.	^28^
Metagenomics (Sequencing of V3-V4 region of the bacterial 16S rRNA)	*Lactobacillus rhamnosus, Enterococcus faecalis*	Determination of the effects of direct-fed microbes on the rumen microbiome of goats	• Showed the influence of direct-fed Lactic acid bacteria on the microbiota in goats	^35^
Metagenomics (Sequencing of V3-V4 region of the bacterial 16S rRNA)	*L. rhamnosus* PT9, *L. rhamnosus* PT10	Determination of the effects of direct-fed microbes on the rumen microbiome of sheep	• Revealed the effects of the lactic acid bacterial administration on the ruminal microbiome of sheep	^36^
Metagenomics (Sequencing of V3-V4 region of the bacterial 16S rRNA)	*Saccharomyces cerevisiae* boulardii CNCM I-1079 *Lactobacillus acidophilus* BT1386	Determination of the effects of directly fed microbes on calves’ ruminal microbiome	• Decreased in pathogenic and increased in beneficial bacterial populations, respectively. • Impacts on pathways include cell cycle, bile secretion, proteasome, cAMP signaling, thyroid hormone synthesis, and dopaminergic synapse pathways.	^34^
Transkingdom network analysis	*Lactobacillus johnsonii* and *Lactobacillus gasseri*)	Determination of probiotics on the liver functions	• Attenuation of western diet-induced diabetes through the improvement of lipid metabolism and enhanced mitochondrial health.	^45^
Transcriptomics	*Lactiplantibacillus plantarum* LIP-1	Determination of *Lactiplantibacillus plantarum* LIP-1 responses to different Ph	• Unraveled the microbe’s responses and enhanced the means to improve survival in a lyophilized state.	^46^
Transcriptomics	*Bifidobacterium breve* UCC2003	Influence of *B. breve* UCC2003 on the intestinal barrier	• Described the functions of *B. breve* UCC2003 in intestinal epithelial homeostasis during early life in neonatal murine intestinal cells.	^47^
Metatranscriptomics	yeasts and *Lactobacillus species*	The interplay of the microbes during the fermentation of	• Revealed the role of the probiotic microbes in pyruvate metabolism	^49^
Metatranscriptomics	Oral microbiota	Interactions of the oral microbiome in biofilm biomass	• Unraveled the complex interactions of the oral microbiome in biofilm assembly	^50^
Peptidomics and Metagenomics	*Lactobacillus helvetius* NS8 and *Lactobacillus fermentum* NS9	Determine the effects of the probiotics on the peptidome of specific pathogen-free mice	• Alters the hippocampus peptidome by acting on the gut-brain axis. • No substantial alteration on the gut microbiome	^58^
Shotgun metaproteomics	Gut microbiota	Revealed the response of the host to the microbiota	• Revealed human proteins and antimicrobial peptides	^60^
Metabolomics and 16S RNA sequencing	*Lactobacillus casei* Zhang	Determined changes in the metabolic profile of *L. casei* Zhang culture	• Revealed changes in several metabolic pathways including amino acid and carbohydrate metabolism. • Showed the metabolic changes associated with glucose restriction to strain.	^69^
Metabolomics	*Lactobacillus plantarum* MLK 14-2, *L. plantarum* KCCM 11322	Determined the variations in the metabolic changes in kimchi during fermentation with different strains	• Revealed the applications of metabolomics to monitor the fermentation characteristics of the strain. • Showed that the metabolites vary with the starter culture strain used	^71^
Metabolomics	*Lactobacillus plantarum* NCU116	Determined the effects of ingesting *L. plantarum* NCU116 on the metabolite profile of hyperlipidemic rat model fed high fat diet	• Revealed biomarkers associated with high-fat diet in the serum of the model experimental animal. • Showed the biological pathways and functions associated with the metabolites. • Improvement in the hyperlipidemic condition of the rat via biosynthetic and metabolic pathways.	^75^
Metabolomics	*Lactobacillus paracasei* subsp. paracasei SM20 and *Propionibacterium jensenii* SM11	Studied the antiyeast activities of the strains against *Candida pulcherrima* and *Rhodotorula mucilaginosa*	• Antiyeast compounds resistant to proteinase K and pronase E treatments were detected. • Other low molecular weight compounds that play role in the complex antiyeast activities were also identified	^76^
Metabolomics	*Lactobacillus rhamnosus* GG	Determine the effects of supplementing *L. rhamnosus* GG as a probiotic in alcoholic liver disease induced rats	• Normalized the level of fatty acids in the liver and feces of alcoholic liver disease induced rats. • Prevents alcoholic liver disease in the treated rats. • Modifies the gut microbiome to stimulate the synthesis of long chin fatty acids. • Elevates the level of some essential amino acids through amino acid biosynthesis.	^77^
Lipidomics coupled with RT-PCR gene expression	Multi-strain probiotics VSL#3 and IT-3 (containing different strains of lactobacilli, *Bifidobacterium* and *Streptococcus thermophilus*)	Determine the lipidemic response of *Caenorhabditis elegans* to different probiotics preparations.	• Variation in the lipid contents *C. elegans* fed VSL#3 compared to IT-3. • Positive correlation between the genes encoding the fatty acid and the levels of the respective lipids.	^82^
Lipidomics	*Lactobacillus plantarum* APsulloc 331261	study the extracellular vesicle phospholipid of the strain	• Variation in the phospholipid level between the extracellular vesicle and the parent cells • Revealed high level of phosphatidylcholine synthase and lipopolysaccharide which could be used as biomarkers for clinical applications	^83^

The invention of high throughput metagenomic sequencing has aided the analyses of microbes, including non-culturable bacteria, at strain levels^31^. Metagenomics and metatranscriptomics have improved the development of new generations of probiotics. These techniques have also increased our understanding of the human microbiome and its contribution to gut physiology by reducing disease risks^32^. An important aspect of microbiome study is the use of metagenomics to generate testable hypotheses on the mechanisms of disease evasion by the host^33^. In a study to analyze the effects of directly fed microbes on the rumen microbiome of cattle, Fomenky et al.^34^ applied 16S RNA gene sequencing of the V3 to V5 region of the entire microbial community to decipher the effect of *Saccharomyces cerevisiae boulardii* CNCM I-1079 (SCB) and *Lactobacillus acidophilus* BT1386 (LA)), and an antibiotic growth promoter (ATB) on the gut microbiota. These authors further showed the roles of the direct-fed microbial culture on the gut microbial structures with consequent inhibition of pathogens through metagenomics sequencing. The mechanisms of the host-microbe interactions, including effects on pathways like cell signaling, bile secretion, proteasome, cAMP signaling, thyroid hormone synthesis, and dopaminergic synapse pathways, were elucidated^34^ (Table 1). Similarly, Maake et al.^35^ and Mani et al.^36^ showed the influence of direct-fed microbes on the microbiome of goats and sheep, respectively, and the resultant modulation of the host health (Table 1). Furthermore, Mani et al.^36^ revealed that probiotics could improve the diversity of the gut microbiome while decreasing the level of pathogenic microbes such as *Pseudomonas species*. Maake et al.^35^ also showed the inhibition of pathogenic microbes, including *Chlamydia species*, in goat’s rumen by direct-fed lactic acid bacteria (LAB) using metagenomics analysis. The advancement in genomic sequencing technology has greatly facilitated research in microbial ecology, microbial interactions within the commensal community, and host-probiotics-microbiome interactions through 16S rRNA and shotgun-metagenomic sequencing^37–39^. These studies indicate the potential of metagenomic analyses to interpret microbial structures and diversity in their ecological niches. They revealed the interactions between probiotics on the microbiome and the host^37,40^.

### Transcriptomics and metatranscriptomics

Transcriptomics studies the whole ‘transcriptome’ of a cell, tissue, organ, or organism under defined conditions^41^. The term transcriptome, first attributed to Charles Auffray, is regarded as the whole set of ribonucleic acid (RNA) expressed in a cell, tissue, or organism^42^. Transcriptomics includes everything relating to RNAs, such as transcription and expression levels, functions, locations, trafficking, and degradation^41^. It also encompasses the structures of transcripts and their parent genes regarding starting sites, 5′ and 3′ end sequences, splicing patterns, and post-transcriptional modifications^43^. Transcriptome covers all types of transcripts, including messenger RNAs (mRNAs), microRNAs (miRNAs), and different types of long noncoding RNAs (*lnc*RNAs)^41^. It employs advanced methods to analyze the expression of multiple transcripts under different physiological or pathological conditions, thereby rapidly expanding the understanding of the relationship between the transcriptome and phenotypes across a wide range of living entities^41^. RNA sequencing data has enabled the quantification of gene expression, non-coding RNAs (ncRNAs), and post-transcriptional regulations in an organism^44^.

Gene expression studies coupled with electron microscopy by Rodrigues et al.^45^ showed the ability of *Lactobacillus johnsonii* and *Lactobacillus gasseri* strains to modulate the activity of the liver with resultant improvement in lipid metabolism through enhancing mitochondrial health in type-2 diabetes-induced mice models. Transcriptomics was used to study the internal response of cells or organisms to physical perturbations. For example, Jingjing et al.^46^ applied transcriptomics analysis to unravel the responses of *Lactiplantibacillus plantarum* LIP-1 to different pH conditions. These authors revealed the mechanisms of survival of the organism in a lyophilized state. Transcriptomics has also been applied to decipher host-microbe interactions in understanding the influence of probiotics on host immune modulation. Kiu et al.^47^ described the effects of *Bifidobacterium breve* UCC2003 in strengthening the intestinal barrier by modulating the intestinal epithelial cells. They showed the impact of the microbiome on the intestinal epithelial cells through global RNA sequencing, differentially expressed genes and metabolic pathways. These authors further revealed the central role of *B. breve* UCC2003 in maintaining intestinal epithelial homeostasis in neonatal murine intestinal cells.

Metatranscriptomics is a powerful tool for studying the microbial community’s structure and transcriptional regulation of active genes through gene expression in response to environmental perturbations^48^. Like metagenomics, metatranscriptomics has been applied to reveal microbial structure and diversity from different ecological niches. Song et al.^49^ described the interplay between the fermentation bacteria during solid-state fermentation in producing Chinese Mao-Tai-flavored liquor using high-throughput 16S rRNA gene amplicon sequencing, internal transcribed space amplicon sequencing, and metatranscriptomics sequencing. These authors showed the involvement of yeasts (genera *Pichia*, *Schizosaccharomyces*, *Saccharomyces*, and *Zygosaccharomyces*) and lactic acid bacteria (genus *Lactobacillus*) in pyruvate metabolism through metatranscriptomics analysis. Similarly, the complex interactions of the oral microbiome in biofilm assembly were elucidated by Edlund et al.^50^ using metatranscriptomics analysis. Apart from applying metatranscriptomics in studying microbial structure through gene expression, this high throughput technique was also used to screen targets in disease conditions such as bacterial vaginosis^51^. Metatranscriptome analysis normally sequences the entire transcriptome from the microbial community and unravels the complex interactions between probiotics, the microbiome, and the host. It can give a clear picture of specific targets and precision application of probiotics, especially when coupled with other omics techniques such as proteomics and metabolomics.

### Proteomics and metaproteomics

The proteome is the total protein complement of a cell or subcellular fraction of an organism in a specified growth phase and physiological condition^52,53^. Proteomics allows the identification of proteins that participate in cellular processes such as catalysis and stress responses and quantifying the complete proteins present in a cell and tissue under defined conditions^53^. Proteomics permits high-throughput identification of important proteins for probiotic interactions with their environment, including the food and host’s gut^53^. The application of proteomics also provides a unique framework for identifying post-translational modifications (methylation, phosphorylation, or glycosylation) that may strongly affect protein functions commonly overlooked by other “omics” techniques^54^. Currently, there is an increase in the amount of proteomics data in public repositories, which has continued to grow at an unprecedented rate^55,56^. Proteomics has been combined with metabolomics to characterize the functions of probiotics originating from different means of production and determine the influence of production processes on the probiotic’s potential^57^. A previous study employing low molecular weight proteomic analysis (peptidomics) by Zhang et al.^58^ revealed the ability of probiotics to modulate the gut-brain axis through a dynamic landscape of the peptidome across multiple regions. This study indicates the potential of proteomics to unravel probiotic interactions with the host beyond the gut to the nervous system.

In-depth Characterization of the microbiome composition, structure, and functions is usually achieved by sequencing the total DNA and RNA^59^. However, the gene/transcripts do not necessarily denote complete protein expression. Therefore, metaproteomics which measures the total expressed proteins, has been considered to provide exact functional information^60^. Additionally, mass spectrometry (MS) based proteomics allows for the simultaneous measurement of proteins of both the host and microbiome^61^ and is a useful tool that aids in unraveling host-microbe interactions in complex intestinal ecosystems^62^. Verberkmoes et al.^60^ demonstrated metaproteomics as an important tool in understanding the host’s response to the gut microbiota by revealing thousands of proteins, including antimicrobial peptides. Thus, showing the mechanisms of the complex interplay between the host and the microbiome. These studies indicate the applications of proteomics in understanding probiotics interactions with the gut microbiome and the host’s responses to such modulations. Metaproteomics is a powerful tool for functional microbiome analysis and can reveal complex host-microbe interactions^61^. Metaproteomics focuses directly on the total proteins expressed in a microbial community and therefore provides an understanding of the community phenotypes^63^. Additionally, proteins constitute the bulk of a cell; hence metaproteomics estimates the contribution of individual community members to the community biomass^64^.

### Metabolomics and lipidomics

Metabolomics is an important technique that simultaneously detects hundreds of small molecules in a biological system^65,66^. The metabolites reveal the organism’s health by acquiring information about the biological system and providing a clear picture of the phenotype^67^. Metabolomics identifies the putative substances from probiotics, such as reduced glutathione, beneficial to the host^45^. Metabolomics quantitively and qualitatively analyze low molecular weight compounds, including peptides, carbohydrates, amino acids, nucleic acid metabolites, vitamins, organic acids, and minerals produced by microbial metabolism^68^. A previous study combining 16S rRNA gene sequencing with proton nuclear magnetic resonance analysis revealed that metabolomics correlates specific biological functions with taxonomy, providing an understanding of the mechanisms underlying the inhibition of pathogens by some probiotic bacteria^69^. Moreover, studies have revealed that bioactive secondary metabolites produced by many probiotic agents affect bacterial community interaction and attenuate the virulent markers on several pathogens^70^. Metabolomics has also been applied in studying the changes that occur during the fermentation of foods and dairy products in the presence of lactic acid bacteria^71–74^. Several studies have applied metabolomics to elucidate metabolic changes resulting from the administration of probiotic bacteria, such as the alleviation of hyperlipidemia in rats by *Lactobacillus plantarum* NCU116^75^, Characterization of antimicrobial metabolites by *Lactobacillus* and *Propionibacterium* coculture^76^, alleviation and the prevention of alcohol-induced liver disease in experimental rat models^77^, and the determination of probiotic function through metabolic Characterization^78^. Metabolomics can be used in probiotics to identify the precise bioactive substances with specific functions for desirable applications, thereby aiding precision therapy. Lipidomics encompasses a broad range of mass spectrometry (MS) workflows that aim to identify and quantify various lipid classes, including their molecular species in the biological systems^79,80^. Lipidomic technologies become useful to characterize the lipid content of an organism through the analysis of the structure, function, or interaction of cellular lipids, which also play an essential role in nutritional research^81^. Lipidomics has been used to show probiotics’ impacts on the host’s physiology. A recent study combining gene expression and lipidomics (using mass-spectrometry) revealed the response of *Caenorhabditis elegans* (*C. elegans*) to multi-strain probiotics; VSL#3 and IT-3 (containing four lactobacilli, three bifidobacterial, and *S. thermophilus* strains) with a resultant variation in the level of the fatty acids based on dietary interventions^82^. The lipidomic analysis of the extracellular vesicles of *Lactobacillus plantarum* APsulloc 331261 probiotic revealed a variation in the phospholipid contents secreted in the media compared to that of the parent cell. Hence giving an insight into the mechanism of lipid biogenesis and lipid-mediated cell-to-cell interactions between or within species^83^. Considering the powerful applications of lipidomics in understanding the biochemistry of the cell membrane^84^, integrating lipidomics data with other ‘omics’ techniques would be a great means of understanding probiotics functions and their influence on the host and gut microbiome.

## The need for integrative ‘multi-omics’ in studying biological systems

A single ‘omics’ technique is insufficient to understand biological processes clearly. For instance, metagenomics sequencing alone is fundamentally limited due to its inability to account for the functional activities of the microbial community directly. Individual omics techniques/data provide important information in understanding several biological processes. However, each type of study (genomics, transcriptomics, proteomics) neglects the influence of the other domains and their interplay. Hence, resulting in limited information on the biological processes^85^. Furthermore, the collective studies and integration of omics data from the host and microbiome domains (a concept known as holo-omics) are imperative for understanding the mechanisms of host-microbe interactions^86^. While multi-omics employs data from one domain (host or microbe), holo-omics integrates data from the host and the microbiome. For instance, to accurately model the health-associated outcomes of bacterial configuration, omics datasets covering several covariables from the host and microbial domains are integrated to reveal the nature of interactions^87^. As a result, additional omics data are needed for a full description of microbial activities, such as the abundances of RNA (metatranscriptome), proteins (metaproteome), and metabolites (metabolome), preferably in an integrated fashion^88^.

**Fig. 1:**
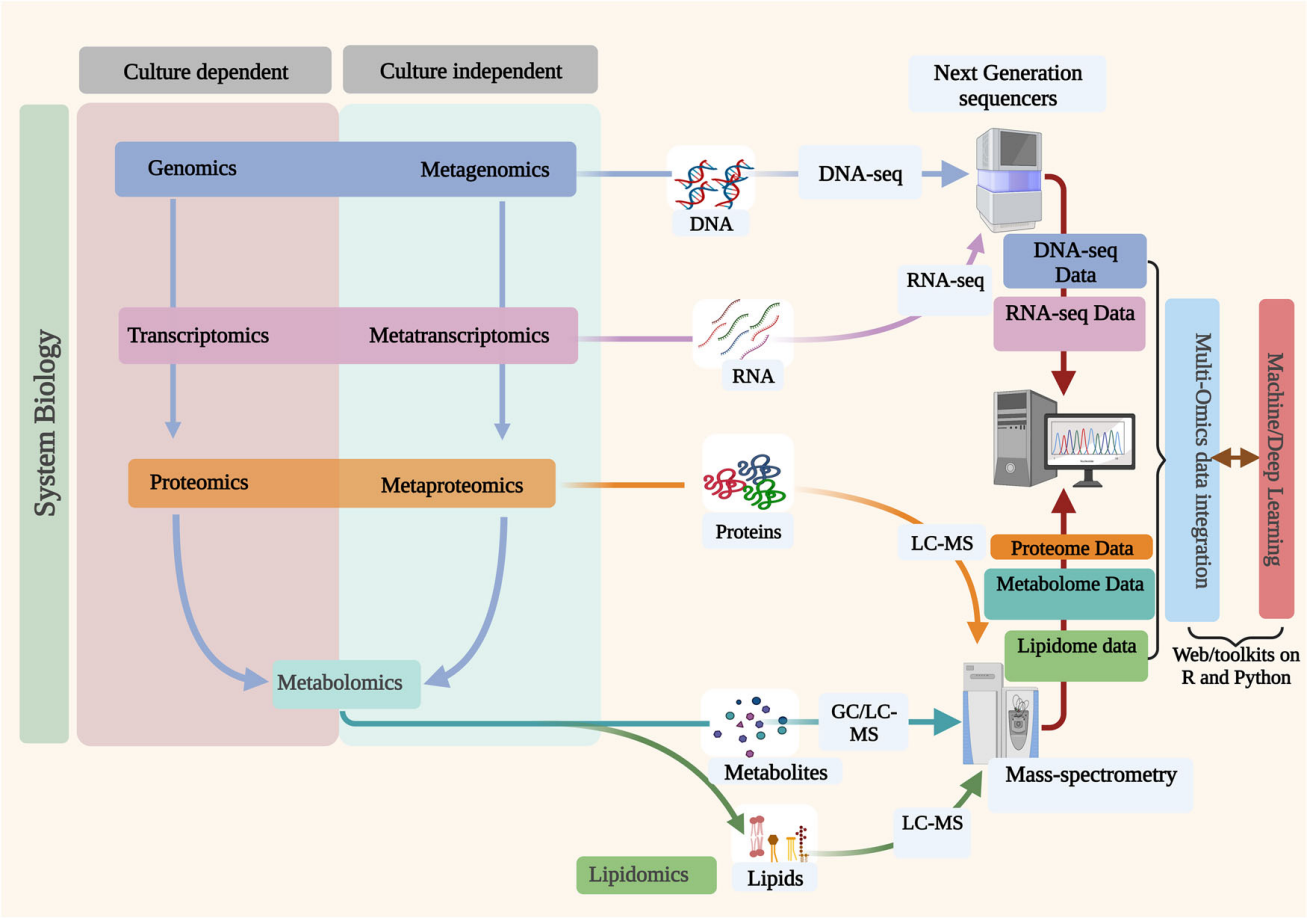
Multi-omics applications in studying biological systems. Omics techniques have applications in both culture-dependent and culture-independent studies of bacteria. The data generated from each technique can be integrated for a holistic understanding of the biological system.

Integrative multi-omics has also paved the way for understanding complex disease interplay and gives a clear route for applying therapeutic regimens^89^. The growing interest in the relationships between the host and their associated microorganisms has changed the perceptions of the biology underlying the host’s genetic properties based on their interactions in several biological processes^85^. These microbes play important roles in the host, including nutrient acquisition^90^, immune modulation^91^ and development^92^, biomolecule synthesis^93^, and influence on the host’s behavior^94^. Furthermore, the advent of meta-omics techniques such as metagenomics^95,96^, metatranscriptomics^97^, and metaproteomics^98^ enables understanding of microbial behaviors in their natural habitat where they are part of communities frequently dominated by as-yet unculturable populations^99,100^. A summary of the different omics techniques applicable for both culture-dependent and culture-independent microbial studies and the other various biological levels for multi-omics data integration for system biology is presented in Fig. 1.

### Multi-omics application at different biological levels and domains in probiotics and microbiomics

Studying the host genomics revealed the host genome’s influence on the diversity of the microbiome and how it affects the host phenotypes^101^. Similarly, metabolomics reveals the metabolic activities of the gut microbiome and its influence on the host gut metabolome^102^. Hence, an integrative multi-omics approach employing metagenomics and untargeted metabolomics of the host and microbiome at both the intra- and inter-domain levels revealed the host-microbiome metabolites interactions with insight into the effects of microbiota on ageing^102^. Furthermore, genomics aids in mining probiotic potentials from commensal microbes^103^, while metagenomics can decipher probiotics-microbiome interactions^104^. Integration of transcriptomics, proteomics and metabolomics datasets profiles host-microbe crosstalk and the influence of probiotics supplementation on the host^105^. The immunomodulatory characteristics of probiotics are determined using transcriptomics by applying RNA-sequencing or microarray gene expression. The proteome changes are determined using protein chips with antibodies, nucleic acid or other proteins that bind to protein targets^106^. The application of multi-omics enables the purification of antimicrobial peptides from microbes using liquid-chromatography tandem mass-spectrometry and the characterization of the genes encoding these compounds through integrated genomics and proteomics analysis^107^. Thus, enabling an evolutionary insight using comparative genomics and discovering novel antimicrobial compounds. This is because mass-spectrometry can identify antimicrobial peptides sequence and masses from a sample mixture, revealing their primary, secondary, and tertiary structures and functions^108,109^.

### ‘Omics’ data integration to elucidate probiotics action

Biological data integration describes the analytical methods that combine information from multiple sources into a single biological inference^110^. Multi-omics data integration provides information on biomolecules from different biological layers for the systematic and holistic understanding of complex biology^22^. Integrated approaches combine individual omics data sequentially or simultaneously to elucidate the complex interplay of molecules^111^. This help in assessing information flow from one omics level to the other, thus closing the gap from genotypes to phenotypes^21^. Approaches to profile cellular characteristics and processes from the genome^112^, epigenome^113^, RNA^114^, RNA isoforms^115^, and proteins^116^ are increasingly applied for understanding cellular activities, especially in single-cell studies^117^. Multi-omics data integration combines individual omics datasets sequentially to understand molecular interactions^111^. Analyzing two or more datasets is necessary for understanding the relationships between different biological functional levels. It is becoming evident that the integration of ‘omics’ data, such as transcriptomics, proteomics, and metabolomics, provides a better understanding of the biological system^21,118^. Recent work by Lee et al.^119^ revealed the integration of metagenomics, genomics, and transcriptomics of bacteria and the analysis of mouse intestinal transcriptome and serum metabolome data to show the mechanisms by which bacteria determine the efficacy of cancer therapeutics where *Bifidobacterium bifidum* (a probiotic bacteria) was found to be influential in patients responsive to therapy. Their result further showed the ability of *B. bifidum* to potentiate the production of interferon-γ through the enhancement of immune-stimulating molecules and metabolites. A longitudinal multi-omics integration of data from the gut microbiome, metabolome, host epigenome, and transcriptome of the mechanisms behind irritable bowel syndrome (IBS) revealed purine metabolism as a novel host-microbial metabolic pathway in IBS with translational potential^120^. Another study by Rasmussen et al.^121^ applied 16 S metagenomic sequencing and untargeted metabolomics to reveal probiotics’ immunomodulatory and growth-promoting actions in rainbow trout. Their studies further elaborated on the association of the gut microbial diversity with the microbial metabolites when fed the probiotics preparation. Hence, revealing the complex mechanisms of microbiome-host interactions. Studies highlighting multi-omics applications in understanding probiotic actions, especially in-vivo, are rare. However, several studies have shown the applications of integrative multi-omics in host-microbiome interactions^85,122–124^. Therefore, the need for integrative multi-omics studies in understanding the actions of probiotics on the host and the host microbiome is crucial.

## Integrative ‘multi-omics’ data platforms and tools

Previous studies have revealed large-scale improvements in data coverage and measurement fidelity to track dynamic changes in RNA transcripts, ribosome profiling, proteins, and metabolites quantitatively in unprecedented detail^125–127^. Multi-omics studies provide the potential for a more holistic picture enabling a comprehensive understanding of complex diseases and biological processes^128,129^. This has led to increased bioinformatics and statistical tools to aid the integration of multiple omics datasets^130^. A previous study by Huang et al.^131^ described several tools that integrate multi-omics datasets for deriving useful biological inferences. Therefore, more recently developed platforms, including web tools for multi-omics data integration, are discussed in this study. A study by Zhou et al.^132^ introduced OmicsAnalyst (https://www.omicsanalyst.ca/), a web-based platform for integrating and visualizing multi-omics data. OmicsAnalyst is user-friendly and aided by three main visual analytic tracks: feature correlation network, cluster heatmap, and dimension reduction analyses. OmicsAnalyst supports three multi-view clustering algorithms: spectral and perturbation clustering and similarity network fusion. Another multi-omics data integration platform is the PaintsOmics 3 (https://www.paintomics.org/), a free web-based interphase that allows integrated analyses of multi-omics datasets, visualization, and network analysis onto the KEGG pathway^133^. PaintsOmics is interactive in its usage and permits extensive exploration of multi-omics data in addition to comprehensive pathway analysis, automatic feature name/identifier conversion, multi-layered feature matching, pathway enrichment, network analysis, interactive heatmaps, trends charts, and accepts a variety of omics data types including transcriptomics, proteomics and metabolomics, and region-based approaches such as ATACseq or ChIP-seq data. The increase in the need for a comprehensive understanding of biological systems has prompted the development of integrative network analysis platforms like OmicsNet^134^ (https://www.omicsnet.ca/) and MOPED^135^ (Multi-Omics Profiling Expression Database, http://moped.proteinspire.org), which are also web-based multi-omics platforms. OmicsNet allows data integration from different sources, including humans and the microbiome and is enhanced with several network analytics functions and interactive 3D charts. MOPED was designed to enhance the integration of genomic and protein expression data into pathway analysis. It also has meta-data detailing functions to ensure data quality, consistency, and reuse^136,137^.

In conclusion, this article reviewed the applications of different omics techniques in studying probiotics and the gut microbiome in culture-dependent and culture-independent scenarios. The need for a systematic approach to understanding the biological system also motivated this work to highlight the integration of multi-omics data in studying probiotics and the gut microbiome. In this study, we elaborated on the applications of different multi-omics data integration platforms available for studying probiotics and gut microbiomes. The different multi-omics data integrating platforms applicable in the study of probiotics and the gut microbiome have the potential to integrate and interpret ‘omics’ data from different biological levels and take various impute data sources. The studies of probiotics at the different biological levels, and the integration of the derived omics data, can unravel the important trends in their activities and the specific mechanisms by which they exert their health benefits. Hence, the room for careful selection and applications in precision therapy.

### Reporting summary

Further information on research design is available in the Nature Research Reporting Summary linked to this article.

## Supplementary information

Reporting summary
